# The Preliminary Results of Bortezomib Used as A Primary Treatment for An Early Acute Antibody-Mediated Rejection after Kidney Transplantation—A Single-Center Case Series

**DOI:** 10.3390/jcm9020529

**Published:** 2020-02-15

**Authors:** Aureliusz Kolonko, Natalia Słabiak-Błaż, Henryk Karkoszka, Andrzej Więcek, Grzegorz Piecha

**Affiliations:** Department of Nephrology, Transplantation and Internal Medicine, Medical University of Silesia, 40-027 Katowice, Poland; nataliablaz@gazeta.pl (N.S.-B.); hkarkoszka@poczta.fm (H.K.); awiecek@sum.edu.pl (A.W.); g.piecha@outlook.com (G.P.)

**Keywords:** acute humoral rejection, first-line therapy, outcomes, proteasome inhibitor

## Abstract

Proteasome inhibitor bortezomib has been used in the treatment of refractory cases of acute and chronic antibody-mediated rejection (AMR) in kidney transplant recipients. However, its efficacy and safety as a primary treatment for early AMR has been scarcely investigated. We herein present our preliminary experience with bortezomib- and plasmapheresis-based primary treatment for early AMR. Thirteen patients transplanted between October 2015 and September 2019 were treated (starting at median 19th post-transplant day) with bortezomib/plasmapheresis protocol for early biopsy-proven AMR. Twelve out of thirteen patients received 4 doses and one patient recieved 3 doses of bortezomib (1.3 mg/m^2^ per dose). In 11/13 patients, 4–7 concomitant plasmapheresis sessions were performed, with or without intravenous immunoglobulin (IVIG). Of note, rituximab was not used in all study patients. The kidney graft and patient survival were 100%. The mean 3-month estimated glomerular filtration rate (eGFR) was 55.3 (95%CI: 44.9–65.8) mL/min/1.73m^2^, 8/13 patients completed 12-month follow-up with mean eGFR 60.4 (45.4–75.4) mL/min/1.73m^2^, and 6/13 patients completed a 24-month follow-up period with mean eGFR 73.9 (56.7–91.1) mL/min/1.73m^2^. Neutropenia < 1 G/L was observed in one patient, third or fourth grade thrombocytopenia in two patients, and eleven patients needed a blood transfusion (median: 2 units/patient). The mid-term results of a primary bortezomib-based treatment for kidney AMR showed its non-inferiority as compared to preceding regimens and acceptable safety. However, our data should be validated in a multicenter randomized trial.

## 1. Introduction

Acute antibody-mediated rejection (AMR) accounts for 20–30% of all acute rejection episodes after kidney transplantation and is often associated with poor allograft survival [1]. It rarely occurs in unsensitized patients, but may occur in up to 50% of highly sensitized recipients [2,3]. Diagnosis of AMR is based on the histopathologic features of the graft biopsy (glomerulitis, arterial-transmural lesions, thrombotic microangiopathy, etc.) and the presence of donor-specific antibodies (DSAs), with or without positive C4d staining [4]. Nowadays, most frequently used treatment modalities for AMR include plasmaphereses (PF) or immunoadsorption (IA), intravenous immunoglobulin (IVIG), anti-T-cell therapy (antithymocyte globulin, ATG), and anti-B-cell therapy (rituximab). Unfortunately, long-term results of such therapy remain suboptimal [5,6]. The rationale for PF or IA is the removal of readily available antibodies and, therefore, limiting the acute tissue damage. Currently, PF is the standard-of-care for the treatment of AMR despite the evidence uncertainty [6].

As the plasma cells are the main source of antibody production, the proteasome inhibitor, bortezomib (Velcade^®^, Milennium Pharmaceuticals, Cambridge, MA, USA), had been introduced into the AMR treatment, mostly as a rescue therapy in refractory cases [7,8,9,10]. In general, kidney graft outcomes were significantly better in acute than chronic AMR [8,10,11,12]. In some reports, such an adjuvant therapy was effective in decreasing DSAs and stabilizing kidney graft function in mid-term observation [7,9]. Nonetheless, the rate of graft loss during longer follow-up periods was high and the excretory function of still-functioning transplanted organs was markedly decreased [7,9,13,14]. As early as 2010, Walsh et al. reported the first use of bortezomib along with a single dose of rituximab in two patients as a primary therapy in early post-transplant AMR, with a rapid DSAs elimination and excellent renal function at five and six months of observation [15]. Later on, Waiser et al. compared the effect of bortezomib- versus rituximab-based AMR therapies, but both study subgroups contained acute and chronic AMR cases with a substantial age difference between groups [16]. Since then, only a few case reports have been published [17,18]. The existing patophysiologic evidence of the potential mechanisms leading to the therapeutic effect of bortezomib indicates its ability for causing apoptosis of antibody-producing plasma cells, blocking the secretion of class IgG antibodies against human leukocyte antigens (anti-HLA) [19], and decreasing the number of plasma cells within the graft [20]. In order to maximize the bortezomib efficacy in AMR treatment, in all previously reported protocols, each dose was administered after plasmapheresis session, which aimed to decrease the amount of circulating antibodies. Therefore, we herein present the largest-to-date cohort of kidney transplant recipients with early AMR diagnosed in graft biopsy and primarily treated with bortezomib-based therapy without concomitant rituximab administration. 

## 2. Methods

### 2.1. Study Group

We present a retrospective observational study, including all consecutive kidney graft recipients (KTRs) from our center, transplanted between October 2015 and September 2019 and diagnosed with early biopsy-proven AMR. All patients received their organs from deceased donors and all had negative complement-dependent cytotoxicity (CDC) crossmatch performed immediately prior to transplantation. Flow cytometric crossmatches were not performed. In our country, only patients with negative CDC crossmatch are listed for the transplant centre in order to choose the recipients of organs procured from the donor. Based on the routine pre-transplant screening and the last Luminex results, the presence of DSA with mean fluorescence intensity (MFI) ≥ 5000 eliminates the potential kidney transplant candidate from the ongoing procedure. A routine kidney graft early protocol biopsy was introduced at our center in the second half of 2015. Since than, all patients who were diagnosed with AMR were then assigned to primary therapy, which included bortezomib. The study was conducted in accordance with the Declaration of Helsinki. As this drug is not registered for the AMR therapy, the Bioethic Committee of the Medical University of Silesia was consulted and all patients gave their informed consent for the off-label use of bortezomib in their therapy. Notably, the guidelines for immunosuppressive therapy after kidney transplantation, issued by the Polish Transplant Society, have allowed the use of bortezomib in KTRs with AMR since 2012 [21]. 

### 2.2. Immunosuppressive Protocol 

The standard immunosuppressive protocol included tacrolimus 2 × 0.1 mg/kg twice daily (with target through level 7–12 ng/mL) and mycophenolate mofetil 750 mg twice daily, both started immediately prior to operating procedure, and steroids, starting with the dose of 500 mg of methylprednisolone intravenously (i.v.) during the operation. Induction therapy was based on the rabbit antithymocyte globulin (rATG) (Thymoglobuline^®^, Genzyme Europe B.V., Amsterdam, Holland) in immunologically high-risk recipients (maximum panel reactive antibodies (PRA) titer > 25% and/or the presence of pre-transplant DSAs) or the anti-interleukin 2 receptor blocker basiliximab (Simulect^®^, Novartis Europharm Europe, Dublin, Ireland). Pre-transplant anti-HLA antibodies were evaluated by solid-phase assays using bead arrays and a Luminex platform. Pre- and post-transplant DSAs were determined using a single-antigen bead assay and results were expressed as mean fluorescence intensity (MFI). Among 4 patients who did not receive rATG induction, in one of those patients, the pre-transplant DSA were undetermined and IL-2RB was given, one patient did not received rATG or IL-2RB induction due to the lack of information concerning the presence of DSA at the time of transplantation, and 2 others had their DSA only in HLA class I and in a relatively low titers (i.e., 1992 and 541), so IL-2RB was used. Each administration of rATG was preceded by metyloprednisolone 125 mg i.v., paracetamol 1.0 g i.v., and an antihistaminic drug, and the first dose was started preoperatively and then continued with intermittent dosing based on lymphocyte count. Additionally, routine fluconazole (100 mg), valgancyclovir (labeled dose adjusted to the kidney graft function), and sulfamethoxazole-trimethoprim (2 × 480 mg) prophylaxis was given in rATG-treated patients.

### 2.3. Primary Treatment Protocol of AMR

After diagnosis, bortezomib (four doses, each 1.3 mg/m^2^) was administered subcutaneously the day after the PF session. Concomitantly, 4–8 PF sessions were performed every second/third day, with a plasma exchange rate of 2.0–2.5× patient’s plasma volume. In patients who did not receive rATG induction, the routine valgancyclovir and sulfamethoxazole-trimethoprim prophylaxis was started prior to bortezomib therapy. Additionally, antibiotic prophylaxis with piperacillin/tazobactam was started and continued during the bortezomib/plasmapheresis therapy. Immediately before every subcutaneous administration of bortezomib, above i.v. metyloprednisolone, paracetamol, and an antihistaminic drug were given as a premedication.

### 2.4. Kidney Graft Function and Protocol Biopsies

Kidney graft estimated glomerular filtration rate (eGFR) was calculated based on MDRD (Modification of Diet in Renal Disease) formulation at the 3rd, 12th, and 24th post-transplant month. 

Kidney graft protocol biopsies were performed usually at the 8th–11th post-transplant day. All biopsies were evaluated by one experienced pathologist according to the revised Banff classification [4,22,23,24]. Each kidney biopsy specimen was routinely stained for hematoxyllin and eosin, PAS, Masson trichrome, and silver methenamine. Additionally, SV40 antigen staining specific for polyoma BK virus infection and von Kossa staining for the presence of calcium-phosphate deposits within the tubular lumen or interstitium were performed. We also analyzed histologic signs of potential calcineurin inhibitor nephrotoxicity. Immunohistochemistry was routinely performed (CD4, CD8, CD20, CD68, and C4d) and described semi-quantitatively based on the grade of infiltration (as scattered cells, foci, clusters, groups, or diffused infiltration). AMR was diagnosed based on the following criteria: (1) the presence of histologic signs of microvascular injury (glomerulitis, peritubular capillaritis (PTC-itis), arteriitis, acute tubular injury/necrosis, (2) positive C4d staining, and (3) presence of DSAs. During the follow-up period, control protocol biopsies were performed and analyzed in 9 patients. 

### 2.5. Statistics

Statistical analysis was performed using the Statistica software (StatSoft Polska, Cracow, Poland). Values were presented as means with 95% confidence interval (CI) or medians with Q25–Q75 quartile values. The comparison of kidney graft function before and after bortezomib treatment was performed using the Student’s *t*-test. *p*-values below 0.05 were considered as statistically significant. 

## 3. Results

### 3.1. Baseline Characteristics

Thirteen KTRs (7 males and 6 females) with early acute AMR treated with bortezomib-based primary therapy were analyzed. Their demographic and clinical characteristics are presented in Table 1. The mean recipient age was 53 years (minimum 30, maximum 68), mean body mass index (BMI) was 26.4 (95%CI: 24.0–28.7), and the median dialysis vintage before transplantation was 43 (IQR: 27–64) months. The history of previous blood transfusions was positive in nine, negative in one, and unknown in two patients. Out of six females, four reported past pregnancies. Only three patients had historical and two patients had the last pre-transplant panel-reactive antibodies (PRA) ≥ 25%, whereas nine patients presented positive results of virtual PRA, calculated based on the Eurotransplant Reference Laboratory HLA database version 2.0. Pre-transplant DSAs were present in twelve patients, with median MFI 10,706 (IQR: 2741–11,415) (Table 2). Induction therapy was used in twelve patients, including rATG in nine and basiliximab in three patients, respectively. 

### 3.2. AMR Diagnosis and Treatment

**Figure 1:**
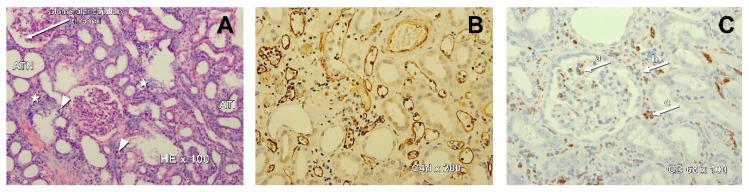
Microphotograph presenting typical histologic findings in kidney transplant recipients with early antibody-mediated rejection. (**A**) Hematoxylin and eosin, magnification 100×. Interstitial edema (tubules are not back to back) with inflammatory infiltrates—asterisks, acute tubular injury (ATI) with the flattening of epithelium cells with the absence of brush border, acute tubular necrosis (ATN) with the tubular basement membranes denuded of epithelial cells, peritubular capillaritis (PTC-itis)—arrowheads, and hypoperfused glomeruli with microthrombi—arrow. (**B**) A diffused C4d staining pattern around peritubular capillaries. Magnification 200×. (**C**) Immunostaining demonstrating CD68-positive macrophages within glomeruli (a), interstitial space (b), and peritubular capillaries (c). Magnification 100×.

AMR was diagnosed based on the first protocol biopsy, performed at a median 10 (IQR: 9–10) post-transplant day (Table 2). Due to technical constraints, the histopatologic biopsy results were available after 2–5 working days. In the majority of patients, the CD4, CD8, and CD68 infiltration was predominantly seen, whereas CD20 staining revealed only single cells or scattered foci, except in three patients, in whom, CD20 clusters and/or groups were described (Figure 1). Hence, a primary AMR treatment started at median 19th post-transplant day. In eight patients, all three Banff criteria of AMR were fulfilled. In the next four patients, the suspected diagnosis was C4d-negative AMR. In one patient, results of pre-transplant single-antigen bead assay were not available, but the screening test for class II anti-HLA antibodies was positive (Table 2). Immediately after AMR diagnosis, the primary treatment was started as described above. One patient received only 3 out of 4 planned doses of bortezomib due to the observed gastrointestinal side effects. In the first three patients, PF treatment with fresh frozen plasma (FFP) was completed. In the next eight patients, FFP together with 5% human albumin was used, in the 1:1 volume proportion. The last two patients were treated with a modified protocol (bortezomib, ATG with total dose 5 mg/kg and IVIG single dose 1g/kg) as they initially received basiliximab induction, whereas we were not able to plan and perform PF sessions at that time. Besides, dialysis therapy was required in five patients before and partially also during the AMR treatment. 

### 3.3. Kidney Graft Function and Survival

**Figure 2:**
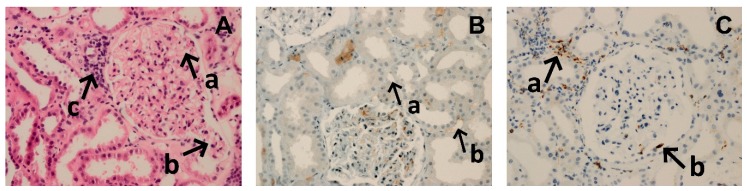
Microphotograph presenting histological findings after bortezomib-based therapy of acute antibody-mediated rejection. (**A**) Hematoxylin and eosin, magnification 200×. Patent capillary lamina without signs of microangiopathy or glomerulitis (a). Prolapse of capillary tuft into the lumen of proximal tubule (b). Small foci of interstitial inflammatory infiltrates (c). (**B**) Negative (a) and nonspecific (b) peritubular capillary C4d staining. Magnification 200×. (**C**) Small interstitial foci of CD68+ cells (a). Single CD68+ cells within glomeruli (b). Magnification 100×.

After bortezomib-based primary AMR therapy, kidney graft function improved in all patients (serum creatinine concentration decreased from mean 4.6 (2.6–6.6) mg/dL before to 1.6 (1.3–1.9) mg/dL after treatment; *p* < 0.001). Mean 3-month serum creatinine concentration (S_Cr_) was 1.35 ± 0.3 mg/dL (eGFR 55.3, 95%CI: 44.9–65.8 mL/min/1.73m^2^), 8/13 patients completed 12-month follow-up with mean S_Cr_ 1.2±0.3 mg/dL (eGFR 60.4, 95%CI: 45.4–75.4 mL/min/1.73m^2^), and 6/13 patients completed a 24-month follow-up period with mean S_Cr_ 1.0 ± 0.2 mg/dL (eGFR 73.9, 95%CI: 56.7–91.1 mL/min/1.73m^2^) (Table 2). In post-treatment control protocol biopsies (Figure 2), four patients presented normal histology, one patient showed the partial resolution of microvascular inflammation, one patient presented mild signs of acute tubular necrosis, and one specimen was inadequate for histopatologic diagnosis. The signs of acute humoral rejection (C4d-) were still present in one patient. One patient was transferred to other transplant center and the results of her control biopsy are unknown. 

In the follow-up period of median 21 (IQR: 6–30) months, both patient and kidney graft survival was 100%. Post treatment DSAs were determined in nine patients and were absent in three of them. Median MFI was significantly lower (1373 (IQR: 0–3046)) than prior to treatment. 

### 3.4. Treatment Safety

Despite the preceded induction therapy with rATG in nine patients treated with bortezomib/plasmapheresis AMR protocol approximately 2–3 weeks later, the treatment was generally well tolerated. In one patient, the last dose of bortezomib was cancelled due to gastrointestinal toxicity. Additionally, in one patient, due to the serious hemorrhage after each of the first two PF sessions and the need of reoperation due to large hematoma, the next six PF had to be performed using citrate to avoid heparin administration. In one patient, we observed ascites of unkonwn reason, which resolved thereafter, and in another, the nasal ulceration was noticed. One patient developed a urinary tract infection during AMR treatment, and another patient presented a reccurrent upper respiratory tract infection within a few post-transplant months, which finally resolved thereafter. Based on the laboratory parameters, neutropenia < 1000 cells/μL was observed in one patient, third or fourth grade thrombocytopenia (<50,000 cells/μL) was observed in two patients, and ten patients needed a blood transfusion (median: 2 units/patient). Finally, in seven patients, the transient mild elevation of liver function tests was noted. No other serious adverse events, including neurotoxicity, opportunistic infections, or malignancy, were observed during the follow-up period. 

## 4. Discussion

In this study, we presented our results of the first-line treatment of early post-kidney transplantation AMR based on the administration of bortezomib accompanied by plasma exchange and/or rATG and IVIG. Those early and mid-term outcomes, quantified by kidney graft function, seem to be adequate, being significantly better than previously reported outcomes in KTRs with the diagnosis of early AMR, in whom the first-line treatment was based on ATG, plasmaphereses, IVIG, and/or rituximab, and then the rescue treatment with bortezomib was applied. Additionally, the overall safety profile during and after the AMR treatment was acceptable. 

The use of proteasome inhibitor in the treatment of early or late AMR was postulated because it targets antibody-producing plasma cells [25]. Notably, the vast majority of AMR episodes are diagnosed during the later post-transplant period, as pre-transplant DSA titers are increasing or de novo DSAs are produced as a consequence of substantial HLA mismatch, immunosuppressive regimen minimization, non-adherence which is increasing over time, and other relevant factors [26,27]. Hence, until now, the main evidence regarding the effectiveness of multimodal AMR treatment in KTRs is based on late AMR episodes, where its success rate is only moderate. Moreover, bortezomib was usually used as a second-line therapy, after the failure of the initial treatment. Regarding its potential utility as a first-line medication, the literature evidence is scarce. To date, after the first report of two cases [15], the same group published the more comprehensive study, including ten patients who received bortezomib-based primary AMR treatment [11]. However, in all these patients, this primary treatment consisted of bortezomib, plasmapheresis, and a single dose of rituximab. The only previous evidence of the sole effect of bortezomib/plasmapheresis as a primary AMR treatment is the comparison of ten patients treated with bortezomib-based regimen with the historic control group of nine patients who received the rituximab-based AMR regimen [16]. The 18-month graft survival was 6/10 in the bortezomib group, much worse than in our present report. However, they diagnosed AMR episodes based on the indication, not protocol biopsies, which may suggest later diagnosis and treatment as compared to our study. Secondly, there were acute and chronic AMR cases mixed together in both groups and the exact post-transplant timing of AMR therapy diagnosis and treatment initiation was not given. Additionally, all PF sessions were performed using only 4% albumin, whereas in our cohort, a fresh frozen plasma constituted approximately 50% of total PF exchange volume. These particular differences, especially those involving the timing of bortezomib therapy initiation, may partly explain the considerable difference in outcomes of kidney grafts after an AMR episode.

We decided to introduce the protocol based on the four labeled doses of bortezomib associated with the concomitant plasmapheresis sessions. In two patients without plasmaphereses, rATG and IVIG were administered instead. Of note, rixutimab was not used in study patients. At the time, as well as the negative literature concerning the efficacy of rituximab in AMR treatment, we also kept in mind the relatively high cost of our previously used rituximab protocol (approximately $1352 USD/dose in patients with 1.8 m^2^ of body surface). Instead, the cost of bortezomib is negligible (~$44 USD/dose in a given patient). Our present case series results suggest an adequate effectiveness of such protocol. In fact, the mean serum creatinine concentration after 12 and 24 months was just about optimal. Of note, the previously reported kidney graft outcomes after the refractory AMR treatment were noticeably worse [7,9,13,14], with several graft losses and suboptimal kidney graft excretory function. We can only hypothesize that some specific properties of bortezomib may condition its effect in the early AMR. As was shown by Perry et al. [19], bortezomib, but not rATG or rituximab, completely abolished anti-HLA antibody production against all HLA specifities. Besides, it also induced the significant increase of apoptotic plasma cells’ percentage in vitro [19]. It is possible that the removal of circulating antibodies by PF results in a rebound of their production, thereby enhancing the sensitivity to proteasome [28]. It could partly explain the efficacy of our primary approach to early post-transplant AMR, with bortezomib, but not rituximab, given immediately as a first-line agent accompanied by PF. As the 12-month serum creatinine concentration was shown to be a good predictor of the long-term kidney graft survival [29], we may expect that the routine primary AMR therapy including bortezomib could also optimize the long-term results of kidney transplantations in this group of patients. 

When analyzing the safety features, it is worth to notice that the spectrum of potentially bortezomib-related hematologic and gastrointestinal disturbances is generally similar to those observed after rATG induction, which was received by the majority of patients presented in this study. Thus, we may assume that the use of bortezomib did not increase the risk of adverse events in subjects who received both medications. Only one patient with leukopenia after initial rATG induction later presented aggravated leukopenia and neutropenia during the bortezomib/plasmapheresis treatment. Besides, in four patients who were not treated with rATG, but received bortezomib, we observed only mild leukopenia in two, thrombocytopenia in two, and slight elevation of liver enzymes activities in seven. Overall, the observed adverse events spectrum in our cohort was similar to the previously reported abnormalities obtained during the treatment of refractory AMR [7,9]. In patients with multiple myeloma, neurotoxicity often limits the use of bortezomib [30]. In this case series, however, we did not observe neuropathy associated with bortezomib use.

We are aware about the study limitations, namely its retrospective character and the low number of patients. However, we present the largest case series of KTRs with early diagnosed AMR, primarily treated with bortezomib. Heterogeneity of AMR treatment regimen is the another limitation, with 2/13 patients treated without plasmaphereses (but with rATG and IVIG instead), and with plasmaphereses performed in the first three patients only with sole albumin supplementation. Nevertheless, rituximab was not used in these patients, whereas the labeled dose of bortezomib was the core of the primary AMR therapy. Also, the last few patients had a shorter follow-up period and a lack of post-treatment biopsies and assessment of DSAs. Nevertheless, 6/13 patients completed the 24-month and 8/13 completed the 12-month observation period. Finally, the authors considered the lack of control group as a serious limitation. 

In conclusion, the mid-term observation of primary bortezomib-based treatment of early post-kidney transplant AMR showed its non-inferiority as compared to previously proposed regimens and acceptable safety profile. It could encourage clinicians to perform early protocol biopsies, especially in patients with high immunological risk, and initiate such a therapeutic protocol as a first-line treatment. However, our data should be confirmed in a larger, multicenter randomized trial. 

## Figures and Tables

**Table 1:** Baseline demographic and clinical characteristics of kidney transplant recipients with bortezomib-treated antibody-mediated rejection (AMR).

Patient	Gender	Age (years)	Cause of ESRD	KTx No	PRA (%)	PRA max (%)	vPRA Class I	vPRA Class II	vPRA Class I+II	Pre-Treatment DSA (MFI)	Induction	Early Graft Function
1	M	30	GNC	2	50	87	98.108	0	98.108	A2 (3489)	rATG	SGF
2	M	41	Unknown	1	0	15	0	0	0	A2 (15,101)	rATG	SGF
3	M	61	ADPKD	2	0	0	31.732	0	31.732	A1 (1968)	rATG	SGF
4	F	61	PNC	1	0	15	46.798	99.476	99.607	DR 4 (10,284)	rATG	IGF
5	M	68	PNC	1	0	0	n/a	n/a	n/a	n/a	IL-2RB	SGF
6	F	52	GNC	1	0	0	30.335 *	80.568	85.604	DR 13 (1500)	rATG	SGF
7	M	61	GNC	2	0	0	0	41.179	41.179	DR10 (11,509)	None	DGF
8	F	63	GNC	1	25	40	68.006	0	68.006	B18 (11,321)	rATG	SGF
9	F	56	Unknown	1	0	0	12.620	93.770	94.541	A11 (1798)	rATG	DGF
10	M	47	GNC	3	5	70	96.361	42.897	97.977	A68 (13,936)	rATG	DGF
11	F	51	ADPKD	1	0	0	75.197	48.049	86.827	B35 (4899)	rATG	DGF
12	M	47	ADPKD	1	0	0	0	0	0	A2 (1992)	IL-2RB	DGF
13	F	60	DM	1	0	0	0	0	0	A3 (541)	IL-2RB	DGF

* Only Cw antigens. AMR, acute antibody-mediated rejection; ESRD, end-stage renal disease; KTx, kidney transplantation; PRA, panel-reactive antibodies; vPRA, virtual PRA; DSA, donor-specific antibodies; MFI, mean fluorescence intensity; GNC, glomerulonephritis; PNC, pyelonephritis; ADPKD, autosomal dominant polycystic kidney disease; DM, diabetes mellitus; rATG, rabbit antithymocyte globulin; IL-2RB, interleukin-2 receptor blocker; SGF, slow graft function; IGF, immediate graft function; DGF, delayed graft function; n/a, non available.

**Table 2:** Protocol biopsy findings and serum creatinine concentration before primary AMR treatment and during the follow-up period.

Patient	Bx POD	Banff Biopsy Scoring										C4d	Other Diagnosis	DSA	Diagnosis	Serum Creatinine Concentration (mg/dL)				
		(g)	(t)	(i)	(v)	(ah)	(ptc)	(cg)	(ct)	(ci)	(cv)					Pre-Treatment	Post-Treatment	3 Month	12 Month	24 Month
1	10	2	0	0	0	0	2	0	0	0	0	1		1	AMR	5.7	2.2	1.1	1.1	1.1
2	8	2	0	0	0	1	2	0	0	0	0	1		1	AMR	2.5	1.8	1.2	1.3	1.3
3	9	2	0	1	1	0	1	0	1	0	0	1		1	AMR	1.8	0.8	1.1	0.9	0.8
4	10	1	0	0	0	0	1	0	0	0	0	1		1	AMR	0.9	0.7	0.8	1.1	0.8
5	9	2	0	0	0	0	1	0	0	0	0	1		0	AMR susp	1.6	1.3	1.2	1.4	1.3
6	18	0	0	2	0	0	2	0	0	0	0	1		1	AMR	6.2	1.5	0.9	0.9	0.9
7	9	3	0	3	1	0	2	0	0	0	0	0	TCMR	1	AMR susp	12.7	1.5	1.3	1.5	
8	10	0	0	0	1	1	0	0	1	0	0	0	TCMR IIA	1	AMR susp	2.7	1.7	1.5	1.6	
9	10	0	0	0	0	2	0	0	0	1	0	1		1	AMR	5.9	1.7	1.7		
10	10	2	0	0	1	0	2	2	0	0	0	1		1	AMR	8.5	3.0	1.4		
11	8	0	0	0	3	0	2	0	0	0	2	1		1	AMR	3.9	1.9	1.7		
12	11	1	1	2	0	2	2	0	0	0	1	0		1	AMR susp	4.0	1.2	1.9		
13	9	2	0	1	1	0	2	0	1	1	1	0	CNI	1	AMR susp	3.1	1.4	1.4		

AMR, acute antibody-mediated rejection; AMR susp, the suspected AMR; Bx, protocol biopsy; POD, post-operative day; DSA, donor-specific antibodies; TCMR, T-cell mediated rejection; CNI, calcineurin inhibitor toxicity.
